# Integrating the Autistic Experience Into Existing Models for Disordered Eating

**DOI:** 10.3389/fpsyg.2022.926415

**Published:** 2022-06-21

**Authors:** Phaedra Longhurst, Lilli Clark

**Affiliations:** ^1^British Association for Counsellors and Psychotherapists (BACP), Leicester, United Kingdom; ^2^School of Health in Social Science, University of Edinburgh, Edinburgh, United Kingdom

**Keywords:** Autism Spectrum Disorder (ASD), eating behaviour and eating disorder (ED), cognitive-behavioural theory, theoretical model, framework, neurodiversity

## Introduction

Autism Spectrum Disorder (ASD) is a lifelong neurodevelopmental condition characterized by anomalies in two domains: social interactions and communication, and restricted/repetitive patterns of behaviors (American Psychiatric Association [APA], 2013). Cognitive-behavioral processes (e.g., central coherence, set shifting) associated with autism have further been linked with eating disorders (EDs) (Huke et al., 2013): life-threatening psychiatric conditions that affect psychological, physical and social well-being (American Psychiatric Association [APA], 2013; Schmidt et al., 2016). As such, research has established several commonalities in underlying cognitive and neural phenotypes in autism and anorexia nervosa (AN) (characterized by restrictive food intake, disproportionate fear of weight gain and weight and shape concerns; American Psychiatric Association [APA], 2013; Westwood and Tchanturia, 2017). More specifically, evidence suggests autistic traits, such as differing cognitive-behavioral and/or body-sensory processing, increases the risk of developing disordered body and eating pathology (Tchanturia et al., 2013; Vuillier et al., 2020). Extant ED research has, however, approached autism as a co-occurrence with EDs: an afterthought to a primary ED symptom profile. Thus, what remains is the exploration into the opposite and increasingly isolated phenomenon – EDs in people *with* autism (and related neurodiverse markers). Consequently, present screening tools and interventions for EDs lack adaptation and translation to neurodivergent populations (Huke et al., 2013; Tchanturia et al., 2020). Current research and practice therefore inaccurately capture and/or target how constructs are experienced in this population, negatively impacting the rigor and validity of ED intervention development and their application(s) (Nicolaidis et al., 2020).

As part of developing screening tools and interventions, researchers are encouraged to refer to theoretical models to guide their content, design, and administration (Campbell et al., 2000; Skivington et al., 2021). A systematic review identified twenty-three theoretical models for disordered eating, most of which refer to cognitive-behavioral approaches and have helped inform the development of screening tools and interventions for disordered eating (Pennesi and Wade, 2016). Conceptual ambiguity in current theory is, however, maintained by relying on cognitive-behavioral features that often differ in presentation (observed from others) and phenomenology (ones' *felt* experience) among autistic individuals, including: (i) body self-schema, (ii) cognitive biases, (iii) compensatory behaviors, and (iv) psychological risk factors for developing and/or maintaining disordered body and eating pathology (see Williamson et al., 2004). As a result, this perpetuates an axis of contention regarding limited generalisability among individuals with cognitive-behavioral variances, such as autistic individuals (Pennesi and Wade, 2016). Furthermore, existing models lack reference to other EDs commonly associated with autism – in particular, avoidant/restrictive food intake disorder (ARFID) (persistent avoidance/disturbance in feeding or eating due to sensory concerns and/or lack of interest; American Psychiatric Association [APA], 2013; Farag et al., 2021). For a more inclusive conceptualization of EDs, existing models should be adapted, or a parallel explanatory framework should be developed alongside existing practice, to better serve neurodivergent populations.

Here, we synthesize transdisciplinary autism research to better account for presentations distinctive to the neurodiverse population. While it is beyond the scope of this Opinion to yield a simple, concrete model, it will illustrate an actionable opportunity to develop an integrative framework which addresses current limitations in the ED literature by integrating the autistic experience into existing models for EDs.

## Defining Autistic Experience: The Autistic Personality and Cognitive Compensation

Within autism research, the *autistic personality* (AP) consists of behaviors denoting an underlying neurobiological endophenotype identified within the diagnostic profile for autism (Landry and Chouinard, 2016; Sarovic, 2021). The AP conceptualizes autism as being manifold (see Sarovic, 2021), in contrast to the Autism Quotient Questionnaire (AQ) (Baron-Cohen et al., 2001), which originally quantified autistic traits as being unidimensional – that is, the “continuum from ASD to normality” (Murray et al., 2014, p. 56). This notion of *normality* has, in effect, led to current research and practice to compare – and inherently pathologize – AP features against behaviors which are traditionally considered ‘typical' (Sucksmith et al., 2011). The AP, however, shifts the autistic experience away from this reductionist, nosographic sphere by considering primary phenomenon, rather than behavioral epiphenomenon – specifically, it is contextual and idiosyncratic in its approach. Differentiating the “disorder” (ASD) to the AP in the context of EDs is therefore foundational to understanding the unique sequalae of EDs from a neurodiverse perspective, rather than through “normative” observation – for instance, acknowledging adaptive eating behaviors to be autism-specific (e.g., avoiding certain food groups to manage sensory difficulties; Kinnaird et al., 2019), rather than inherently being maladaptive (e.g., avoiding food for weight-loss).

While the AP and AQ both recognize phenotypic issues – in particular, presentations of a maladaptive behavioral phenotype (Sarovic, 2021) – the AP is more complex by accounting for the accumulative effect of behavioral difficulties (e.g., altered sensory processing) subsequent to *cognitive compensation* (CC): the cognitive ability to compensate for, and overcome, maladaptive AP features through learning and carrying out adaptive responses (see Pugliese et al., 2015, 2016; Livingston and Happé, 2017). Although relatively temporally stable, CC is dependent on internal (e.g., heritability, executive function) and external factors (e.g., day-to-day cognitive demands; Happé and Frith, 2006; Livingston and Happé, 2017). CC is therefore associated with a multitude of risk factors, implying diverse potential pathways which may generate impairment and contribute to AP maladaptation (Lenroot and Yeung, 2013). As a result, CC is highly individualized : two individuals may present equal cognitive deficits, however present differences in behavior due to varying in amount of CC; AP features may also lessen over time in one individual, to the extent they no longer fulfill the ASD diagnostic profile (Livingston and Happé, 2017). In other words, the AP presents heterogeneity across dynamic psychic facets, influencing disorder developmental trajectory and outcomes (Uljarevic et al., 2017) – which should be accounted for within ED theory and practice. Moreover, impaired CC increases the probability of the AP to become both pronounced and maladaptive, giving rise to complex mental health difficulties (Geurts et al., 2014; Ullman and Pullman, 2015; Lever and Geurts, 2016). Overall, rather than applying a diagnosis of AN across all samples presenting restrictive eating and/or body dissatisfaction, we argue additional investigation to determine whether such symptomatology is simply features of the AP (e.g., lower interoceptive awareness) or inadequate CC/maladaptive AP (Sarovic, 2021).

## Operationalising the Autistic Experience Into Existing Models of Disordered Eating

Brede et al. (2020) developed a model of restrictive eating in autism, which proposes autistic-specific mechanisms to induce ED development and/or maintenance. This model presents underlying mechanisms and/or presentations through an “autistic” lens, including sensory sensitivities (e.g., altered interoceptive awareness); social interactions and relationships; impaired self and identity; emotional difficulties; and alternative thinking styles. Whilst these features are general risk factors for EDs among the neurotypical population, they disproportionately affect autistic individuals in terms of severity and frequency (Westwood and Tchanturia, 2017). Therefore, it is plausible that beneath the surface of “typical” risk-factors (e.g., appearance internalization) lies autism-specific risk factors (e.g., social camouflaging/masking; Brede et al., 2020).

Features of the (non-pathological) AP acts as a “first-hit” for precipitating ED-like behavior (e.g., missing meals/overeating by accident), as an individual reports differences in cognitive and body-sensory processes, such as altered interoceptive awareness (e.g., differing awareness of hunger, satiation or thirst; see Hatfield et al., 2019; Kinnaird et al., 2019). However, research considers these body-sensory processes as typically “innate,” moderating both positive (e.g., intuitive eating) and negative outcomes (e.g., disordered eating) (Tribole and Resch, 2003). Subsequently, existing models associate these processes with the ED etiology and/or target of intervention. For example, the multidimensional model of AN proposes biogenetic and personality factors (e.g., low interoceptive awareness) to predict ED onset (Lyon et al., 1997), while the acceptance model of intuitive eating posits intuitive and/or adaptive eating to prevent and/or reduce ED symptomatology (Augustus-Horvath and Tylka, 2011; Babbott et al., 2022). Furthermore, the AP and its features can be broadly categorized into two domains: social (e.g., communicative/relational difficulties) and non-social (e.g., repetitive/restrictive behaviors, intense/obsessive interests; see Happé et al., 2006). Yet, current ED practice is predominantly informed by theory, such as the cognitive-interpersonal maintenance model of AN (Schmidt and Treasure, 2006; Treasure and Schmidt, 2013), which again asserts that AP features – particularly, obsessive-compulsive and interpersonal difficulties – act as predisposing and/or maintenance factors for EDs. Overall, existing ED theory and practice assume features of the AP to inherently underlie ED pathology (Table 1 presents a non-exhaustive list which highlights these current conceptual ambiguities). Yet, the incidence of mental health difficulties amongst autistic individuals “might [alternatively] speak to the demanding and taxing nature of [CC]” (Livingston and Happé, 2017, p. 736). Current theory and practice should therefore accommodate – rather than pathologize – the AP, and alternatively focus on the “second-hit” of impaired CC, which precipitates maladaptive responses to AP challenges, such as sensory sensitivities, and manifests ED symptomatology. For example, (re)conceptualizing restrictive eating to include rationales like avoiding sensations of digestion due to sensory sensitivities (Trevisan et al., 2012).

**Table 1:** A list of the main existing constructs and independent variables of disordered eating as summarized by Pennesi and Wade (2016, p. 184) and confounding autistic features.

**Constructs**	**Independent variables**	**Confounding autistic features**
Preoccupation weight and shape	Weight and shape concern, body-image disturbance; body-dissatisfaction; appearance anxiety; body shame	Social Camouflaging/masking; Intense interests
Self-esteem deficits	Low self-esteem; dysphoria; ineffectiveness; poor self-concept; aversive self-awareness	Impaired sense of self; Experiences of stigmatization and marginalization
Emotional regulation difficulties	Mood intolerance; emotional distress; emotional dysregulation; emotional avoidance; emotional eating; affective instability	Self-regulation difficulties; Alexithymia
Interpersonal issues	Family functioning; family connectedness; social dependency; response from close others	Language and communication difficulties; Differing social interaction abilities; Altered theory of mind
Thin-ideal internalization and external pressure	Endorsement of thin ideal; pressure to be thin; pressure to diet; family-peer weight norms and teasing; media, parental and peer influences	Thinking styles (e.g., literal, black and white); Social information processing difficulties
Perfectionism	Cognitive rigidity	Ritualistic behaviors; Need for control and predictability
Cognitive factors	Negative self-belief; negative automatic thoughts; maladaptive cognitions; negative and positive beliefs about eating	Maladaptive phenotype; Unrecognized/undiagnosed ASD
Dieting	Dietary restraint; unhealthy weight control behaviors	Difficulties with sensory processing; Exercising as a form of stimming
Self-surveillance	Poor interoceptive awareness	
Negative affect	Depression; Anxiety	

Existing models are, however, restricted in variances of cognitive processes and related maladaptive behavioral outcomes. This is despite the role of inadequate CC for maladaptive AP, and its plausible influence on the onset of ED-related beliefs and/or behaviors (e.g., appearance investment). For instance, autistic individuals report differing social interaction and communication abilities (the AP) and, when met with the lower ability to adaptively learn from social interactions (lower CC), present secondary disordered body and eating pathology as a maladaptive resource (e.g., changing ones' appearance to “fit in”; Brede et al., 2020). Whilst this might resemble maladaptive behaviors presented in neurotypical groups, such as difficulties with social functioning precipitates a negative sense of self and disordered eating as proposed by the interpersonal model of binge eating (Wifley et al., 2000), CC serves as a “scaffold” for social behavior (socially adapted behaviors which are achieved through conscious CC strategies; Ullman and Pullman, 2015; Livingston and Happé, 2017). For example, autistic individuals may engage in masking/social camouflaging (suppressing AP behaviors) in an attempt to appear neurotypical (Lai et al., 2016; Hull et al., 2017). Although these strategies potentially facilitate social navigation, they are susceptible to rapid decline and/or maladaptation when faced with stressors (e.g., mental fatigue), and are inflexible to novel or ambiguous situations (Hull et al., 2017; Livingston and Happé, 2017). As a result, an individual may “mask” in way which adopts external, socio-cultural influences (e.g., copying anorexic values; Brede et al., 2020). This potentially contextualizes the subtle differences in the ways in which autistic individuals understand and/or experience relevant constructs, such as dieting and self-surveillance (see Kinnaird et al., 2019).

As such, it is conceivable that existing models are limited in experiential and/or construct equivalence among autistic individuals, subsequent to the lack of divergence in neurocognitive mechanisms associated with lower CC – namely, executive function (cognitive abilities in planning, inhibition and cognitive flexibility; see Hill, 2004). For instance, the dual-pathway model for bulimia nervosa (BN) suggests socio-cultural processes (e.g., thin internalization) and environmental factors (e.g., harmful media messaging) promote body dissatisfaction, manifesting ED pathology (Stice, 2001). Whilst this may be the case among autistic individuals, it is, instead, subsequent to presenting deficits in executive function and theory of mind which alters social information processing (e.g., difficulties with interpreting societal messaging) and thinking styles (e.g., literal, black and white thinking; Hill, 2004; Mazza et al., 2017; Kalandadze et al., 2018), manifesting disordered body and eating beliefs – “She takes things as absolutely true and cannot cope with nuances – ‘If I'm not thin then I'm fat and horrible', with nothing in between” (Brede et al., 2020, p. 4289).

Both internal and external sources therefore moderate CC processes and AP maladaptation (Livingston and Happé, 2017), similar to the tripartite influence model of body dissatisfaction and disordered eating (van den Berg et al., 2002; Keery et al., 2004; Yamamiya et al., 2008) which proposes external/environmental factors (e.g., media, family) to promote maladaptation (e.g., thin internationalization), manifesting negative outcomes (e.g., body dissatisfaction, disordered eating). However, what is unique to the autistic experience is *environmental accommodation*, where one's immediate environment may facilitate or impede CC, thereby moderate AP maladaptation (Johnson et al., 2015). More specifically, autistic individuals experience certain environments negatively, as they contain increasing demands (e.g., requires social interaction, sensory overload) which directly induces lower CC and, therefore, AP maladaptation (Livingston and Happé, 2017; Kerr-Gaffney et al., 2020). In turn, this potentially elicits negative emotional consequences (e.g., impacted sense of self, emotional dysregulation) which, as a result, reinforces ED-related behaviors/coping strategies (Mansour et al., 2016; Brede et al., 2020). For example, Kinnaird et al. (2019) found autistic individuals presented ED behaviors as a way of dealing with communication difficulties during social interactions, as well as sensory issues during mealtimes. Although these findings are preliminary, we argue that this highlights nuances in current correlates, predictors and consequences for EDs.

## A (Unifying) Example for Future Endeavors

An impetus for defining and subsequently describe the confounding autistic experience of EDs was to illuminate current gaps in the ED literature. Existing theory remains to lack conceptual variation in cognition and behavior and focuses on a pathological rather an inclusive view of autism, making it difficult to explore the influence of ED-related independent variables (e.g., weight and shape concerns) within this population. We hypothesize that autistic individuals will understand and/or experience theoretical constructs, such as body dissatisfaction and internalization, differently to neurotypical groups. Taken together, this suggests testing the applicability and acceptability of existing models using a mixed methods design. We therefore propose a current, actionable, and accessible framework which enhances inclusive ED theory and practice and follows guidelines for conducting research with the autism community (see Gowen et al., 2019).

As a contextual factor, researchers may test the Acceptance Model of Intuitive Eating (Augustus-Horvath and Tylka, 2011) through four stages. First, scholars may investigate how constructs within a model (e.g., intuitive eating) are experienced among autistic individuals using a qualitative design, both in non-clinical (e.g., Kinnaird et al., 2019) and clinical settings (e.g., Barraclough et al., 2019). Second, translate and validate an existing measure (e.g., Intuitive Eating Scale-2; Tylka and Kroon van Diest, 2013) through psychometric testing (e.g., exploratory to confirmatory factor analysis) among both autistic and neurotypical samples (see Swami and Baron, 2019). This is with the aim to determine reliability and validity – in particular, its idiomatic and experiential equivalence – within this population. In doing so, this addresses the lack of validated measures for autistic individuals and may promote positive outcomes (e.g., reduce mis- or under-diagnosis). As recommended in the MRC framework (Campbell et al., 2000; Skivington et al., 2021), *development* should be carried out prior to systematically developing an intervention. Thus, the third stage involves identifying or developing theory through examining modeling processes and outcomes using structural equation modeling (e.g., Oh et al., 2012). This informs the fourth and final stage, which involves evaluating the efficacy of an existing programme which has been previously found to improve ED outcomes (e.g., Eat for Life; Bush et al., 2014). Alternatively, researchers may develop an adapted intervention which responds to ASD literature. For example, incorporating intuitive eating with enhancing interoceptive task performance among autistic individuals (e.g., ADIE; see Quadt et al., 2021). This should include testing their (i) feasibility (piloting), (ii) efficacy (evaluation), and (iii) effectiveness (implementation) (see Campbell et al., 2000; Skivington et al., 2021). We call for developments to be made with an approach which is sensitive to the autistic experience, by adequately promoting community research involvement (see Pickard et al., 2021) and responding to the ASD literature (e.g., Brede et al., 2020). Overall, we argue this will determine whether current screening tools and interventions effectively translate to this population, however considering cognitive and behavioral variances; and/or developments specific to this population are necessary.

## Conclusion

The autistic experience of EDs receives little theoretical or empirical investigation, despite autistic individuals presenting poorer and long-enduring outcomes (Westwood and Tchanturia, 2017). Autism-ED research is limited in acknowledging the nuances of the autistic experience in two ways: first, research lacks accounting for its heterogenic nature more generally and/or its entirety (e.g., development, presentation); second, despite it not being limited to a specific ED diagnostic category and/or severity level, research predominantly refers to restrictive eating pathology (Brede et al., 2020). Furthermore, existing ED theory has yet to be extended to feeding disorders such as ARFID, despite its prevalence among both neurotypical and neurodiverse groups – particularly children and adolescents (Nicely et al., 2014; Farag et al., 2021). Consequently, current understanding limits engagement and advancement of neurodiverse detection and intervention practices. By drawing inferences from autism-ED literature, this Opinion highlights how the autistic experience confounds existing models of EDs: the AP is be expressed in a way which resembles typical ED symptom profile; however, it is, instead, maladaptive AP features and impaired CC (or cognitive ability) which manifest ED pathology. As this is likely to be relevant to other neurodiverse phenotypes (e.g., ADHD), the potential utility of reframing existing models for EDs from a neurodiverse perspective may ultimately advance research and clinical practice concerning heterogeneity, and enhance inclusive assessment, prevention and treatment applications. This Opinion therefore aims to serve as a catalyst for future development, as we call for research and practice to integrate the autistic experience into existing models for EDs through an integrating framework.

## Author Contributions

PL and LC contributed to writing and editing the manuscript. Both authors contributed to the article and approved the submitted version.

## Conflict of Interest

PL was employed by the British Association for Counselling and Psychotherapists. The remaining author declare that the research was conducted in the absence of any commercial or financial relationships that could be construed as a potential conflict of interest.

## Publisher's Note

All claims expressed in this article are solely those of the authors and do not necessarily represent those of their affiliated organizations, or those of the publisher, the editors and the reviewers. Any product that may be evaluated in this article, or claim that may be made by its manufacturer, is not guaranteed or endorsed by the publisher.
